# AutoCloner: automatic homologue-specific primer design for full-gene cloning in polyploids

**DOI:** 10.1186/s12859-020-03601-7

**Published:** 2020-07-16

**Authors:** Alexander Coulton, Keith J. Edwards

**Affiliations:** Biological Sciences Department, The University of Bristol, 24 Tyndall Avenue, Bristol, BS8 1TQ UK

**Keywords:** PCR, Primer-design, Polyploid, Gene-cloning, Crops Wheat

## Abstract

**Background:**

Polyploid organisms such as wheat complicate even the simplest of procedures in molecular biology. Whilst knowledge of genomic sequences in crops is increasing rapidly, the scientific community is still a long way from producing a full pan-genome for every species. Polymerase chain reaction and Sanger sequencing therefore remain widely used as methods for characterizing gene sequences in many varieties of crops. High sequence similarity between genomes in polyploids means that if primers are not homeologue-specific via the incorporation of a SNP at the 3’ tail, sequences other than the target sequence will also be amplified. Current consensus for gene cloning in wheat is to manually perform many steps in a long bioinformatics pipeline.

**Results:**

Here we present AutoCloner (www.autocloner.com), a fully automated pipeline for crop gene cloning that includes a free-to-use web interface for users. AutoCloner takes a sequence of interest from the user and performs a basic local alignment search tool (BLAST) search against the genome assembly for their particular polyploid crop. Homologous sequences are then compiled with the input sequence into a multiple sequence alignment which is mined for single-nucleotide polymorphisms (SNPs). Various combinations of potential primers that cover the entire gene of interest are then created and evaluated by Primer3; the set of primers with the highest score, as well as all possible primers at every SNP location, are then returned to the user for polymerase chain reaction (PCR). We have successfully used AutoCloner to clone various genes of interest in the Apogee wheat variety, which has no current genome sequence. In addition, we have successfully run the pipeline on ~ 80,000 high-confidence gene models from a wheat genome assembly.

**Conclusion:**

AutoCloner is the first tool to fully-automate primer design for gene cloning in polyploids, where previously the consensus within the wheat community was to perform this process manually. The web interface for AutoCloner provides a simple and effective polyploid primer-design method for gene cloning, with no need for researchers to download software or input any other details other than their sequence of interest.

## Background

Cloning of genetic sequences via polymerase chain reaction (PCR) is a routine operation in biological research. In agricultural research specifically, this procedure facilitates the connection between varietal sequence differences and important phenotypic traits such as disease resistance, yield, and abiotic stress tolerance. This process is significantly complicated in polyploid crops due to the presence of multiple closely-related subgenomes, meaning that allele-specific primers must be used to prevent cloning of non-target sequences such as homeologues and paralogues. Although there already exists a tool for designing primers for use in Kompetitive allele specific PCR (KASP) assays in polyploids, PolyMarker [1], this only considers flanking sequences of 100 bases either side of a varietal SNP. This limitation means that it cannot used to clone entire genes, as the mean ± s.d. length of a high-confidence gene in the IWGSC RefSeq v1.0 [2] wheat genome assembly is 3065 ± 3957 bases. There are currently no software packages to assist allele-specific primer design for the cloning of entire genes or other genomic sequences of interest, and indeed current practice within the community is to carry out this lengthy process manually [3–5].

For example, consider the situation in which a researcher has a gene sequence from a single wheat variety and is interested in how this sequence differs between varieties. To assess this, they could design several pairs of allele-specific primers whose products overlap, covering the entire gene region, and then sequence these products after performing PCR. This primer design process involves several stages. First, the wheat genome must be queried for closely related alleles to the sequence of interest. Once homologues have been identified and extracted, they must be arranged into a multiple sequence alignment. This alignment must then be scanned for SNPs to serve as the 3’ bases of primers, which can then be designed using the appropriate primer-design software. In total, this is a lengthy process that would be significantly improved via the use of an automated tool. Here we present AutoCloner, a fully automated allele-specific primer design pipeline that includes a simple web interface for users. Although developed in the context of wheat, AutoCloner can easily be configured to work with any species for which a genome assembly is available. It requires only a single input, the sequence of interest to clone.

## Implementation

AutoCloner first searches for homologues of the user input sequence by performing a BLASTN search of the input sequence against the latest IWGSC (International Wheat Genome Sequencing Consortium) RefSeq wheat genome assembly [2]. Alternatively, AutoCloner can use any genome that the user has specified in the configuration file, and could therefore be used for any species where homologous sequences with high similarity are common. The tabular output files of the BLAST search are parsed and used as a basis for sequence extraction from the genome assembly. BLAST breaks up query sequences into high-scoring pairs [6], and as such it is necessary to examine groups of hits when using BLAST to extract homologues rather than individual hits. Here a group of BLAST hits are defined as hits with the same query and subject sequence that are within 1000 bases of each other. The group of BLAST hits that is most closely related to the input sequence is assumed to be the genomic representation of that sequence and is used to obtain the flanking regions of DNA. The next three, or alternatively the number specified by the user, best groups of hits are also used for sequence extraction and are assumed to be close homologues of the input sequence, providing their within-group average bitscore exceeds 200. This threshold means that only hits with a reasonable amount of sequence similarity are retained. The amount of sequence to extract that flanks the sequence inputted by the user is specified by the start buffer (−s) and end buffer (−e) parameters of the pipeline, which default to 1000 nucleotides. To ensure memory is used efficiently during sequence extraction, AutoCloner makes fasta indices of any genome assemblies that are specified in the configuration file. AutoCloner also has the capability to include more than one genome if there are genome sequences available for more than one variety within the species. If this is the case, one sequence from each of the additional genomes is also extracted to increase the reliability of SNP identification in later stages, ensuring that varietal SNPs are not used as a basis for primer design.

The extracted sequences must then be arranged into a multiple sequence alignment, which is used to identify SNPs between the sequence of interest and its homologues. AutoCloner uses Muscle [7], or alternatively Dialign [8], to achieve this. Dialign is useful when multiple homologues and partial homologues, i.e. sequences of different lengths, are present in the sequence set extracted from the BLAST results, as it allows specification of anchors that inform the alignment. A single-nucleotide mismatch at the 3’ end of the primer significantly decreases the efficacy of Taq polymerase in the PCR reaction [9], and so the SNP locations can be evaluated as potential primer locations. It is unlikely that each of these locations will have the ideal sequence characteristics for a primer, such as adequate GC content, low probability of hairpin structures and an ideal melting temperature (TM). It is therefore common for a researcher to evaluate multiple locations before finding one that is adequate for a primer. This is time consuming and can also produce sub-optimal results due to human error.

**Fig. 1:**
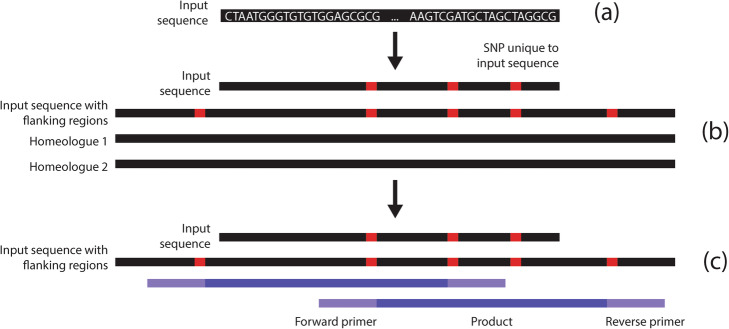
Schematic detailing AutoCloner, a homologue-specific primer design pipeline for polyploids. **a** The user inputs a DNA sequence which they wish to clone in a variety for which this sequence is unknown. **b** AutoCloner finds flanking regions and homologues through BLAST, generates a multiple sequence alignment with Muscle and scans the alignment for SNPs. **c** The best possible combination of primers, whose overlapping products span the entire input sequence, are returned by the pipeline via Primer3

AutoCloner evaluates all possible combinations of primers at the SNP locations that fall within the user-specified minimum and maximum product size ranges by passing each combination to Primer3 [10] and using the Primer3 output parameter PRIMER_PAIR_PENALTY to select the best combinations. This process is repeated until enough sets of forward and reverse primers with overlapping products have been selected to cover the entire input sequence (Fig. 1). These overlapping products allow the input sequence to be cloned and sequenced in its entirety. In addition to the primers intended for PCR, several within-product primers are also selected for Sanger sequencing of large products. AutoCloner also allows the user to input their own multiple sequence alignment instead of a single sequence using the -a option; in this case the initial stages of the pipeline are skipped and the alignment is immediately scanned for SNPs. Note that if this is the case, AutoCloner expects the multiple alignment to 1) be in Fasta format with gaps indicated by “- “and 2) for the sequences to be in the following order: sequence to be cloned, same sequence but with flanking regions included, then any homologues.

In order to maximise ease-of-use for wheat researchers, we designed a web interface for AutoCloner using the Django web development framework for Python that integrates into the popular wheat resource website CerealsDB [11]. The web interface requires either a single sequence to be input by the user, or a multiple sequence alignment. Once submitted, the sequence is entered into a queue for processing by AutoCloner. When the pipeline has finished processing the sequence, the user is forwarded to the Details page in which the details of their primers are displayed, with options to download the primer information as a CSV file as well as download the multiple sequence alignment in either fasta or clustal format. The multiple sequence alignment is also displayed directly on the website using MSA [12], a Javascript web component. To maximise ease of use, the website was designed not to require any login details or personal information from the user.

## Results and discussion

It is well established that a DNA mismatch at the 3’ end of the primer significantly reduces the efficiency of Taq polymerase in a PCR. Previous research suggests the magnitude of this effect could as much as a 100-fold decrease [9]. This principle serves as the basis for the design of homologue-specific primers. We used AutoCloner to investigate potential gene candidates underpinning a region of segregation distortion on chromosome 5A of a Chinese Spring X Paragon F5 mapping population [13]. This region lacked distortion in an Apogee X Paragon F5 mapping population [13]. Sequences for this region in both Chinese Spring and Paragon were available, whilst no sequencing data was available for Apogee. We therefore formulated the hypothesis that since there was a lack of distortion in the Apogee X Paragon population, any potential causative gene would have the similar or identical sequences between Apogee and Paragon, and different sequences between Chinese Spring and Paragon. Therefore, sequencing data for Apogee could highlight (or eliminate) genes worthy of further investigation.

**Fig. 2:**
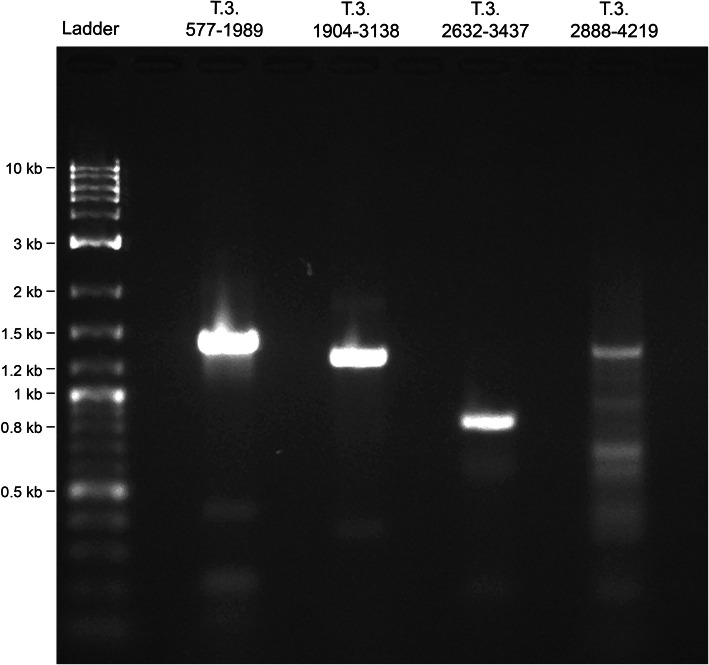
Agarose gels showing amplified PCR products for the TraesCS5A01G531300 gene using primers designed by AutoCloner. Details of the primers are shown in Table 1. The DNA ladder used was the Quick-Load® Purple 2-log DNA ladder, manufactured by New England Biolabs, containing DNA fragments ranging from 0.1 kb to 10 kb in size. The expected product sizes for T.300.577–1989, T.300.1904–3138, T.300.2632–3437 and T.300.2888–4219 were 1412, 1234, 805 and 1331 bases respectively. A subsequent PCR (not shown) in which the annealing temperature was increased from 58 °C to 60 °C increased the specificity of the T.300.2888–4219 set of primers

The first gene we cloned was TraesCS5A01G531300, a 2.4 kb High Confidence gene from the IWGSC assembly [2], with a BLAST search identifying two homeologues on chromosomes 4B and 4D (the result of a well-known translocation between 5A and 4A [14]), as well as a partial homologue on 5B, with homology beginning 325 bases into the gene and extending throughout the gene until ~ 10 bases downstream. Sequence identity, not including regions outside of the HSPs identified by BLAST, between TraesCS5A01G531300 and each of the three homologues was 93, 93 and 80% respectively, whereas GC content for TraesCS5A01G531300 and each of the homologues was 49, 49, 49 and 49% respectively. These GC values are close the average GC percentage of all 110,790 HC genes in the IWGSC assembly [2], which is 51% ± 10 (mean ± s.d). AutoCloner returned four overlapping pairs of primers whose products covered the entire gene length (Table 1). DNA was extracted following the protocol in Edwards et al. [15] . All the primers produced amplicons from the desired locus in the genome (Fig. 2), and the entire gene sequence was obtained via Sanger sequencing of these products. The resulting sequence was identical to the sequence in Chinese Spring apart from one non-synonymous single nucleotide variant.

**Table 1:** List of primers designed by AutoCloner to amplify and sequence TraesCS5A01G531300 in Apogee. Oligo names succeeded by an F are forward primers, whilst an R indicates a reverse primer. Both primers for PCR and primers for Sanger sequencing are included

Oligo Name	Sequence (5′- > 3′)	Type
T.300.577–1989.F	AGACTTCCTGAACACGGCACAA	PCR
T.300.577–1989.R	CTTCTTGATGGCGCGGCATATAT	PCR
T.300.1904–3138.F	TAGGTTGACGTCATCGAAGCAG	PCR
T.300.1904–3138.R	CGGGTGAGAAGCAAGGACTC	PCR
T.300.2632–3437.F	CCATGGTGAGGTTGAGGTCC	PCR
T.300.2632–3437.R	GGTGCAGCAAGAGTACGGAG	PCR
T.300.2888–4219.F	CGGACGATGACAACAGGGAG	PCR
T.300.2888–4219.R	CCCTGCTCTCTCTCTCTCTCT	PCR
T.300.577–1989.split2.F	CTCGAACTCGCTATTGGGCT	Sanger
T.300.577–1989.split3.F	AGTCAAGGTACAATATGTGACTGA	Sanger
T.300.1904–3138.split2.F	CCGTGAAGTACCGAAACCCA	Sanger
T.300.1904–3138.split3.F	TAACGAACCTGGTGCCTTCG	Sanger
T.300.2632–3437.split2.F	TCAGGTCCTTGGCCAGTTTC	Sanger
T.300.2888–4219.split2.F	GTGGAGACATGGAGGAGCAC	Sanger
T.300.2888–4219.split3.F	ACCAACACTCAAGCAAAGGGA	Sanger

The second gene cloned in Apogee was TraesCS5A01G531700.1, a 3.6 kb gene on chromosome 5A with homeologues on 4B and 4D, as well as a partial 2 kb paralogue on chromosome 5A, and smaller 600 bp homologous regions on 2D and 5D. We obtained a complete sequence of the coding region (introns defined by pairwise alignment to the IWGSC gene model) using four sets of overlapping primers produced by AutoCloner. All the amino acid substitutions present between the Chinese Spring and Paragon coding sequences of TraesCS5A01G531700.1 are also present between the Apogee and Paragon sequences. The Apogee sequence also contains some additional substitutions with Paragon between positions 35–69 (Table 2).

**Table 2:** Amino acid substitutions between Paragon and Chinese Spring coding sequences of TraesCS5A01G531700.1. AA = Amino acid; “first variety” refers to the first variety listed in the corresponding “Comparison” column for each row

Comparison	Position	AA in first variety	AA in second variety
Paragon & Chinese Spring	270	M	T
Paragon & Chinese Spring	292	Q	P
Paragon & Chinese Spring	552	I	V
Paragon & Chinese Spring	731	P	H
Paragon & Chinese Spring	760	A	V
Paragon & Apogee	35	L	F
Paragon & Apogee	64	F	V
Paragon & Apogee	66	E	K
Paragon & Apogee	69	E	A
Paragon & Apogee	270	M	T
Paragon & Apogee	292	Q	P
Paragon & Apogee	552	I	V
Paragon & Apogee	731	P	H
Paragon & Apogee	760	A	V
Chinese Spring & Apogee	35	L	F
Chinese Spring & Apogee	64	F	V
Chinese Spring & Apogee	66	E	K
Chinese Spring & Apogee	69	E	A

Also cloned was TraesCS5A01G530800, a 551 bp gene on chromosome 5A. The gene was cloned using a single set of flanking primers produced by AutoCloner, and the gene sequence was found to be identical between Apogee, Paragon and Chinese Spring. This sequence had homologues that encompassed the entire gene on chromosomes 5D, 4B and 4D, as well as 17 small sequences of around ~ 250 bases with high similarity to the flanking region downstream of the input sequence.

In addition to the sequences evaluated in the context of segregation distortion, AutoCloner was also run using all high-confidence gene sequences from the IWGSC assembly under 10,000 bases, amounting to a total of 85,040 genes. These alignments and primer sets are available to view on the AutoCloner website. For 30,186 of the genes, the top two homologous sequences identified by the pipeline were homeologues from the corresponding subgenomes and chromosomes (e.g. for an input sequence on 3A, sequences from 3B and 3D were most closely related). Also of interest was the composition of these alignments in terms of number of regions, not strictly limited to homeologues, that contained high sequence identity to the input sequence. The mean ± s.d. number of these highly similar sequences, detected via BLAST HSPs, was 10.4 ± 7.47 per alignment. When limited to sequences that covered over 70% of the input sequence, flanking regions of 1 kb upstream and downstream included, this number reduced to 2.4 ± 2.24 per alignment, indicating that the majority of genes only have a few close homologues that extend over large regions. The smaller regions with high sequence identity should not be problematic for allele-specific cloning if they do not fully encompass the PCR product. Even so, the AutoCloner web interface includes a “Guided Mode”, allowing the user to manually inspect alignments and remove (or retain) sequences before SNP calling and primer design should these regions be of interest.

## Conclusions

Whilst the scientific community has made incredible progress in producing genomic sequences for many different crop species, we are a long way from having a complete pangenome encompassing every single variety within each species. Until this time, cloning of genes will remain an important technique for assessing genetic variation, and AutoCloner makes this process significantly faster and easier than current methods.

## Availability and requirements

Project name: AutoCloner

Project home page: http://www.autocloner.com

Operating system(s): Linux

Programming language: R

Other requirements: Muscle, Dialign, BLAST, primer3, Biostrings, optparse, tibble, dplyr

License: GNU GPL

Any restrictions to use by non-academics: None

## Data Availability

The AutoCloner web interface is hosted at www.autocloner.com. The source code can be found at https://github.com/alexcoulton/autocloner. An archived version (1.0) of the AutoCloner pipeline described in this manuscript can be found at the DOI: 10.5281/zenodo.3886651. The raw data supporting the findings in this manuscript can be requested from the corresponding author at acoulton.re@gmail.com.

## Abbreviations

BLAST: Basic Local Alignment Search Tool
PCR: Polymerase chain reaction
TM: Melting temperature
SNP: Single-nucleotide polymorphism
License: GNU GPL
None: Any restrictions to use by non-academics
